# Reentry via high-frequency pacing in a mathematical model for human-ventricular cardiac tissue with a localized fibrotic region

**DOI:** 10.1038/s41598-017-15735-5

**Published:** 2017-11-10

**Authors:** Soling Zimik, Rahul Pandit

**Affiliations:** Centre for Condensed Matter Theory, Department of Physics, Indian Institute of Science, Bangalore, 560012 India

## Abstract

Localized heterogeneities, caused by the regional proliferation of fibroblasts, occur in mammalian hearts because of diseases like myocardial infarction. Such fibroblast clumps can become sources of pathological reentrant activities, e.g., spiral or scroll waves of electrical activation in cardiac tissue. The occurrence of reentry in cardiac tissue with heterogeneities, such as fibroblast clumps, can depend on the frequency at which the medium is paced. Therefore, it is important to study the reentry-initiating potential of such fibroblast clumps at different frequencies of pacing. We investigate the arrhythmogenic effects of fibroblast clumps at high- and low-frequency pacing. We find that reentrant waves are induced in the medium more prominently at high-frequency pacing than with low-frequency pacing. We also study the other factors that affect the potential of fibroblast clumps to induce reentry in cardiac tissue. In particular, we show that the ability of a fibroblast clump to induce reentry depends on the size of the clump, the distribution and percentage of fibroblasts in the clump, and the excitability of the medium. We study the process of reentry in two-dimensional and a three-dimensional mathematical models for cardiac tissue.

## Introduction

Reentry occurs in cardiac tissue when a wave of electrical activation, which mediates muscle contraction in a mammalian heart, abnormally reenters the medium instead of propagating normally through cardiac tissue and then being absorbed at its periphery. Such reentry in cardiac tissue has been associated with life-threatening cardiac arrhythmias, like ventricular tachycardia and ventricular fibrillation^1–5^, which are a leading cause of death in the industrialized world (see, e.g.^6^,). Reentry can occur because of many reasons; and all its causes are still not completely understood. The presence of heterogeneities is one such cause; it has been implicated in the initiation of reentry^7–9^, and also known to favour the sustenance of such reentries. It behooves us, therefore, to investigate reentry in cardiac tissue with heterogeneities.

Heterogeneities can exist in a heart because of spatial variations in the electrophysiological properties of the constituent myocyte cells^10^. Such variations can be induced by diseases like ischemia^11–15^, heart failure^10,16^, or genetic disorders like the long-QT syndrome^17–19^. Moreover, heterogeneities can occur because of the presence of other non-myocyte cells, like fibroblasts. Cardiac fibroblast cells, whose electrophysiological properties are different than those of cardiac myocytes, exist naturally in a normal heart and are needed for the proper functioning of a heart: fibroblasts form the extracellular matrix, along with other cells, and, thereby, ensure the structural integrity of the heart^20^. The process of proliferation of fibroblasts in cardiac tissue is known as fibrosis; and it occurs in the wound-healing process after cardiac injuries like myocardial infarction^21^. A large density of fibroblasts, in such diseased hearts^21^, has been shown to be arrhythmogenic^22^.

Experimental investigations have identified the occurrence of fibroblasts over a range of densities. For example, the percentage *p*_*f*_ of fibroblasts has been found to vary from 10–90% in aged rabbit hearts^23^, and from 7–43% in tissues explanted from diseased human hearts^24^. It is important to asses the arrhymogenicity of such fibrotic tissue and its dependence on (a) the organization of the region with fibrosis and (b) the density of fibroblasts. *In silico* studies, which use state-of-the-art mathematical models for cardiac tissue, provide us with a means to investigate systematically the effects of the spatial organization of fibroblasts and the density of fibroblast inhomogeneities, in a background of myocytes^25–31^, on wave propagation through such tissue. Some numerical studies have explored the effects of fibroblasts on electrical-wave dynamics in cardiac tissue^25–31^. However, these references have not investigated systematically how a clump of fibroblasts, in a medium with myocytes, can lead to re-entries; nor have they examined the factors that increase in the propensity of such reentries via high-frequency pacing. We carry out *in silico* studies that we have designed to examine these issues.

As we have mentioned above, regional heterogeneites can occur in cardiac tissue because of diseases like myocardial infarction^32^. Therefore, Alonso, *et al* ^33,34^. have used a simple mathematical model (a model with a few variable) for cardiac tissue, in which they introduce a fibroblast clump, inside which fibroblasts are distributed randomly; they use a single-pulse protocol to show that reentry is most probable when pf is near the percolation threshold. However, in *in-vivo* a mammalian heart is normally under the action of periodic electrical pulses from the Sino Atrial Node (the pacemaker of the heart), or, abnormally, under high-frequency pacing because of conditions like tachycardia. Therefore, it is important to study the arrhythmogenic effects of such fibroblast clumps in cardiac tissue that is stimulated by electrical pulses at different pacing rates.

We go well beyond earlier studies of fibroblast clumps by (a) using state-of-the-art ionic models for cardiac myocytes and fibroblasts and (b) stimulating our simulation domains by pulses whose frequency we vary from low to high values. Our study takes into account the coupling of myocytes and fibroblasts. Although such coupling has not been seen in *in-vivo* experiments, there is evidence of such myocyte-fibroblast coupling in *in-vitro* experiments^35–37^. We study the mechanisms of the occurrence of reentry via pacing in a medium with fibroblast clump. We find that the occurrence of reentry is dependent on the pacing frequency. We first show, by using a persistent pacing protocol, how fibroblast clumps can induce spiral waves via high-frequency pacing, and not at low-frequency pacing. We find that, with such high-frequency pacing, the interaction between the wavebacks and the wavefronts of consecutive waves near the surface of a fibroblast clump can induce wave distortions (WD) that eventually lead to spiral waves. Such WDs and, hence, spiral waves do not occur at low-frequency pacing because of the lack of waveback-wavefront interaction. As the formation of spiral waves depends on the occurrence of WDs, we study the factors that controls the formation of WDs. We find that the average time at which WD occurs depends on the size of the clump, percentage *p*_*f*_ of fibroblasts in the clump, and also on the excitability of the medium. Moreover, apart from the formation of spiral waves because of persistent, high-frequency pacing, we also find that reentry can occur through a different process that does not involve an intermediate step of the formation of WDs. By using a transient pacing protocol, we show that waves can re-enter the medium through the fibroblast clump, and this reentry phenomenon is due to the uneven recovery time of the medium between the regions inside and outside of the clump. Also, we find that occurrence of this reentry is more probable when we pace the medium at high frequency than at low frequency. Lastly, we show that this reentry, via transient pacing, in a three-dimensional tissue occurs at higher *p*_*f*_ as compared to that of in a two-dimensional tissue.

The remaining part of this paper is organized as follows. The Materials and Methods section describes the models we use and the numerical methods we employ to study them. We then describe our results in the section called Results. The final section, titled Discussions, is devoted to a discussions of our results in the context of earlier numerical and experimental studies of the problems we consider.

## Materials and Methods

For our myocyte cell we use the state-of-the-art O’Hara-Rudy model (ORd) for a human ventricular cell^38^ with the modifications implemented in^38^, where the fast sodium current (I_NA_), of the original ORd model, is replaced with that of the model due to Ten Tusscher and Panfilov^39^. This modification is done to set the value of the conduction velocity (CV) of the electrical waves at the experimentally observed values in human cardiac tissue, i.e., 65 cm/s^40^. The original *I*_*Na*_ formalism in the ORd model leads to an unphysiological low value of CV^41^. Furthermore, apart from the primary effects on the CV and the spiral-wave frequency, the modification does not change much the other electrophysiological properties of the original ORd model (we refer the reader to^41^ for a detailed comparison between the original and the modified ORd model). However, the original ORd model and also the ORd model with the modified I_NA_, gives rise to waves with large wavelengths (18 cm); hence, we must use unphysically large tissue size to study the wave dynamics. To overcome this shortcoming, we have reduced the wavelength of the wave by increasing the conductance of outward potassium current (I_Kr_) by two times its original value; and we use this parameter set as the control parameter set for our study. This control parameter set gives a spiral-wave frequency *ω* $\simeq$ 6.4 Hz. The value of *ω* is calculated from the power spectrum of the time-series recording of action potentials from four representative points in our simulation domain. The power spectra of the four time series are averaged; and the value of frequency at which the averaged spectrum shows a dominant peak is taken to be the value of *ω*. Figure S1 in the Supplementary Material shows the four points from where the time series are recorded, a representative time series, and the averaged power spectra.

For our high-frequency pacing we use frequencies that are of the order of *ω*; and for the low-frequency pacing we use a frequency lower than *ω*.

We model fibroblasts as passive cells, for which we use the model given by MacCannell, *et al*.^42^. We model the membrane conductance *G*_*f*_ of the fibroblasts as follows: it has a nonlinear dependence on its membrane voltage *V*_*f*_ as in^43,44^. The value of *G*_*f*_ is set to 1 ns if *V*_*f*_ < −20 mV and to 2 nS for *V*_*f*_ > −20 mV.

In an isolated myocyte, the membrane potential ${V}_{m}$ of the myocyte is governed by the ordinary differential equation (ODE)

1 $$\frac{{{\rm{dV}}}_{{\rm{m}}}}{{\rm{dt}}}=-\frac{{I}_{{\rm{ion}}}}{{C}_{m}},$$

where ${C}_{m}$ is the myocyte membrane capacitance, which has a value of 185 pF; ${I}_{{\rm{ion}}}$, the sum of all the ionic currents of the myocyte is

2 $$\begin{array}{c}{I}_{{\rm{ion}}}={{\rm{I}}}_{{\rm{Na}}}+{{\rm{I}}}_{{\rm{to}}}+{{\rm{I}}}_{{\rm{CaL}}}+{{\rm{I}}}_{{\rm{CaNa}}}+{{\rm{I}}}_{{\rm{CaK}}}+{{\rm{I}}}_{{\rm{Kr}}}+{{\rm{I}}}_{{\rm{Ks}}}+{{\rm{I}}}_{{\rm{K1}}}+{{\rm{I}}}_{{\rm{NaCa}}}\\ \quad \,+\,{{\rm{I}}}_{{\rm{NaK}}}+{{\rm{I}}}_{{\rm{Nab}}}+{{\rm{I}}}_{{\rm{Cab}}}+{{\rm{I}}}_{{\rm{Kb}}}+{{\rm{I}}}_{{\rm{pCa}}};\end{array}$$

a list of all the ionic currents in our model is given in Table 1.

**Table 1:** Table of currents.

I_Na_	fast inward ${{\rm{Na}}}^{+}$ current
I_to_	transient outward ${{\rm{K}}}^{+}$ current
I_CaL_	L-type ${{\rm{Ca}}}^{{\rm{2}}+}$ current
I_Kr_	rapid delayed rectifier ${{\rm{K}}}^{+}$ current
I_Ks_	slow delayed rectifier ${{\rm{K}}}^{+}$ current
I_K1_	inward rectifier ${{\rm{K}}}^{+}$ current
I_NaCa_	${{\rm{Na}}}^{+}{/\mathrm{Ca}}^{{\rm{2}}+}$ exchange current
I_NaK_	${{\rm{Na}}}^{+}{/K}^{+}$ ATPase current
I_Nab_	${{\rm{Na}}}^{+}$ background current
I_Cab_	${{\rm{Ca}}}^{{\rm{2}}+}$ background current
I_pCa_	sarcolemmal ${{\rm{Ca}}}^{{\rm{2}}+}$ pump current
I_Kb_	${{\rm{K}}}^{+}$ background current
I_CaNa_	${{\rm{Na}}}^{+}$ current through the L-type ${{\rm{Ca}}}^{{\rm{2}}+}$ channel
I_CaK_	${{\rm{K}}}^{+}$ current through the L-type ${{\rm{Ca}}}^{{\rm{2}}+}$ channel

The membrane potential ${V}_{f}$ of an isolated fibroblast is governed by the equation

3 $$\frac{{{\rm{dV}}}_{{\rm{f}}}}{{\rm{dt}}}=\frac{-{I}_{{\rm{f}}}}{{C}_{f}},$$

where ${C}_{f}$ = 6.3 pF is the membrane capacitance of the fibroblast, and ${I}_{{\rm{f}}}$ is the fibroblast current

4 $${I}_{{\rm{f}}}={G}_{f}({V}_{f}-{{\rm{E}}}_{{\rm{f}}}),$$

where ${{\rm{E}}}_{{\rm{f}}}$ is the resting membrane potential of the fibroblast, which we set to −30 mV^35–37^.

The various ionic currents incorporated in the ORd model are tabulated above. The symbols used for the currents follow^38^, which gives the dependence of all these currents on the membrane potential and the equations for all the gating variables.

In our model for tissue, with fibroblasts and myocytes, we introduce the fibroblasts (F) randomly between myocytes (M), as in^25,45^. We take into account myocyte-myocyte (MM), myocyte-fibroblast (MF), and fibroblast-fibroblast (FF) couplings. The gap-junctional coupling ${G}_{gap}$, between myocytes and fibroblasts, is set to 8 nS. The myocytes are coupled diffusively with a diffusion constant ${D}_{mm}$ = 0.0012 ${{\rm{cm}}}^{2}$ ${{\rm{ms}}}^{-1}$; and the coupling strength between fibroblasts is set to one-tenth of the MM coupling, i.e., ${D}_{ff}$ = $\frac{{D}_{mm}}{10}$. The fibroblasts are distributed randomly as follows. A site inside the fibroblast clump is occupied with certain probabilities, by a fibroblast or by a myocyte, as follows: The probability ${{\rm{\Omega }}}_{f}$ of a fibroblast occupying a particular site depends on the percentage of fibroblasts as Ω_*f*_ = ${p}_{f}$/100 and, therefore, the probability of a myocyte occupying a particular site is 1-Ω_*f*_.

The formation of infarcted regions, which cause fibrosis, in cardiac tissue are associated with changes in the electrophysiological properties, like remodelling of ion-channel conductances and gap-junctional conductances^46–48^. These electrophysiological changes reduce the conduction velocity of the electrical waves in and around the fibrotic regions. To study the effects of such changes in the wave-velocity on the formation of wave distortions, we only vary the conductance of fast-sodium current ${G}_{Na}$, which is known to occur in infarcted regions^49^. However, to verify our results with a detailed model of fibrotic tissue, we also perform simulations by using a model of fibrotic tissue, where we incorporate the changes in the ion-channel conductances of other currents, apart from ${I}_{Na}$, and also vary the gap-junctional conductances in and around the fibroblast clump; we discuss this model in the Supplementary Material.

The spatiotemporal evolution of the membrane potential in tissue is governed by the equation given below (explicitly for the illustrative case of a two-dimensional square-lattice simulation domain):

5 $$\frac{{{\rm{dV}}}^{{\rm{i}},{\rm{j}}}}{{\rm{dt}}}=-\mathrm{(1}-{\eta }^{i,j})\frac{{I}_{{\rm{ion}}}}{{C}_{m}}-{\eta }^{i,j}\frac{{I}_{f}}{{C}_{f}}+\sum _{ < {\alpha }{\beta } > }{G}_{\alpha ,\beta }({V}^{i+\alpha ,j+\beta }-{V}^{i,j}),$$

where $< {\alpha }{\beta } >$ indicates a sum over the nearest-neighbours of the sites $(i,j)$, i.e., $\alpha$ and $\beta$ can have values +1 or −1. ${G}_{\alpha \beta }$ are the coupling weights to the site $(i,j)$ from its four nearest-neighbour sites. ${G}_{\alpha \beta }$ and ${\eta }^{i,j}$ are given below:

$$\begin{array}{c}{\eta }^{i,j}=\{\begin{array}{c}1,\quad {\rm{i}}{\rm{f}}\,{\rm{s}}{\rm{i}}{\rm{t}}{\rm{e}}\,({\rm{i}},\,{\rm{j}})\,{\rm{i}}{\rm{s}}\,{\rm{a}}\,{\rm{f}}{\rm{i}}{\rm{b}}{\rm{r}}{\rm{o}}{\rm{b}}{\rm{l}}{\rm{a}}{\rm{s}}{\rm{t}}{\rm{;}}\\ 0,\quad {\rm{i}}{\rm{f}}\,{\rm{s}}{\rm{i}}{\rm{t}}{\rm{e}}\,({\rm{i}}{\rm{,}}\,{\rm{j}})\,{\rm{i}}{\rm{s}}\,{\rm{a}}\,{\rm{m}}{\rm{y}}{\rm{o}}{\rm{c}}{\rm{y}}{\rm{t}}{\rm{e}}.\end{array}\end{array}$$

If the site $(i,j)$ is occupied by a myocyte, then

$${G}_{\alpha \beta }=\{\begin{array}{c}\frac{{D}_{mm}}{\delta {x}^{2}},\quad {\rm{i}}{\rm{f}}\,{\rm{s}}{\rm{i}}{\rm{t}}{\rm{e}}\,({\rm{i}}+{\rm{\alpha }},{\rm{j}}+{\rm{\beta }})\,{\rm{is}}\,{\rm{a}}\,{\rm{myocyte}};\\ \frac{{G}_{gap}}{{C}_{m}},\quad {\rm{i}}{\rm{f}}\,{\rm{s}}{\rm{i}}{\rm{t}}{\rm{e}}\,({\rm{i}}{\rm{+}}{\rm{\alpha }}{\rm{,}}{\rm{j}}{\rm{+}}{\rm{\beta }})\,{\rm{i}}{\rm{s}}\,{\rm{a}}\,{\rm{f}}{\rm{i}}{\rm{b}}{\rm{r}}{\rm{o}}{\rm{b}}{\rm{l}}{\rm{a}}{\rm{s}}{\rm{t}}.\end{array}$$

If the site $(i,j)$ is occupied by a fibroblast, then

$${G}_{\alpha \beta }=\{\begin{array}{c}\frac{{D}_{ff}}{\delta {x}^{2}},\quad {\rm{i}}{\rm{f}}\,{\rm{s}}{\rm{i}}{\rm{t}}{\rm{e}}\,({\rm{i}}+{\rm{\alpha }},{\rm{j}}{\rm{+}}{\rm{\beta }})\,{\rm{i}}{\rm{s}}\,{\rm{a}}\,{\rm{f}}{\rm{i}}{\rm{b}}{\rm{r}}{\rm{o}}{\rm{b}}{\rm{l}}{\rm{a}}{\rm{s}}{\rm{t}}{\rm{;}}\\ \frac{{G}_{gap}}{{C}_{f}},\quad {\rm{i}}{\rm{f}}\,{\rm{s}}{\rm{i}}{\rm{t}}{\rm{e}}\,({\rm{i}}+{\rm{\alpha }},{\rm{j}}{\rm{+}}{\rm{\beta }})\,{\rm{i}}{\rm{s}}\,{\rm{a}}\,{\rm{m}}{\rm{y}}{\rm{o}}{\rm{c}}{\rm{y}}{\rm{t}}{\rm{e}}{\rm{.}}\end{array}$$

Note that the FM, FF, and MM coupling constants are different from each other, and, therefore, the value of ${G}_{\alpha \beta }$ varies inside the fibroblast clump.

### Numerical Methods

We use a forward-Euler method to solve the ODEs (1), (3) and (5) and also the ODEs for the gating variables of the ionic currents of the myocyte. The spatial and temporal resolutions are set to be $\delta x$ = 0.02 cm and $\delta t$ = 0.02 ms, respectively. In our two-dimensional (2D) tissue simulations, we use a domain of size ${\rm{512}}\times {\rm{512}}$ grid points, which translates into a physical size of ${\rm{10.24}}\times {\rm{10.24}}\,{{\rm{cm}}}^{{\rm{2}}}$. And, for our three-dimensional (3D) tissue simulations, we use a domain that has ${\rm{384}}\times {\rm{384}}$ grid points in the *x*–*y* plane, and 10 grid points along the vertical $z$ axis. All our tissue simulations are carried out for a duration of 18 seconds. For pacing the tissue, external line stimuli are applied, on the lower boundary of the domain, with strength and duration of −150 $\mu {\rm{A}}/\mu {\rm{F}}$ and $3$ ms, respectively. We use two pacing protocols for our study: (1) the persistent-pacing (PP) protocol and (2) the transient pacing (TP) protocol. In the PP protocol, we apply periodic pulses, of a particular cycle length, persistently until the end of our simulation. In the TP protocol, we apply pulses for a transient period of time (we apply 20 pulses of a particular cycle length), and we stop the pulses after that.

## Results

We first discuss the phenomenon of the formation of wave deformations, which lead eventually to spiral or scroll waves, by using the PP protocol in a medium with a clump of fibroblasts. We also study the factors that affect the formation of such wave deformations. Then, by using the TP protocol, we show how waves can also reenter the medium via the fibroblast clump because of the uneven recovery period between the regions inside and outside of the clump.

### Wave distortions and spiral waves near the periphery of a fibrotic region

**Figure 1:**
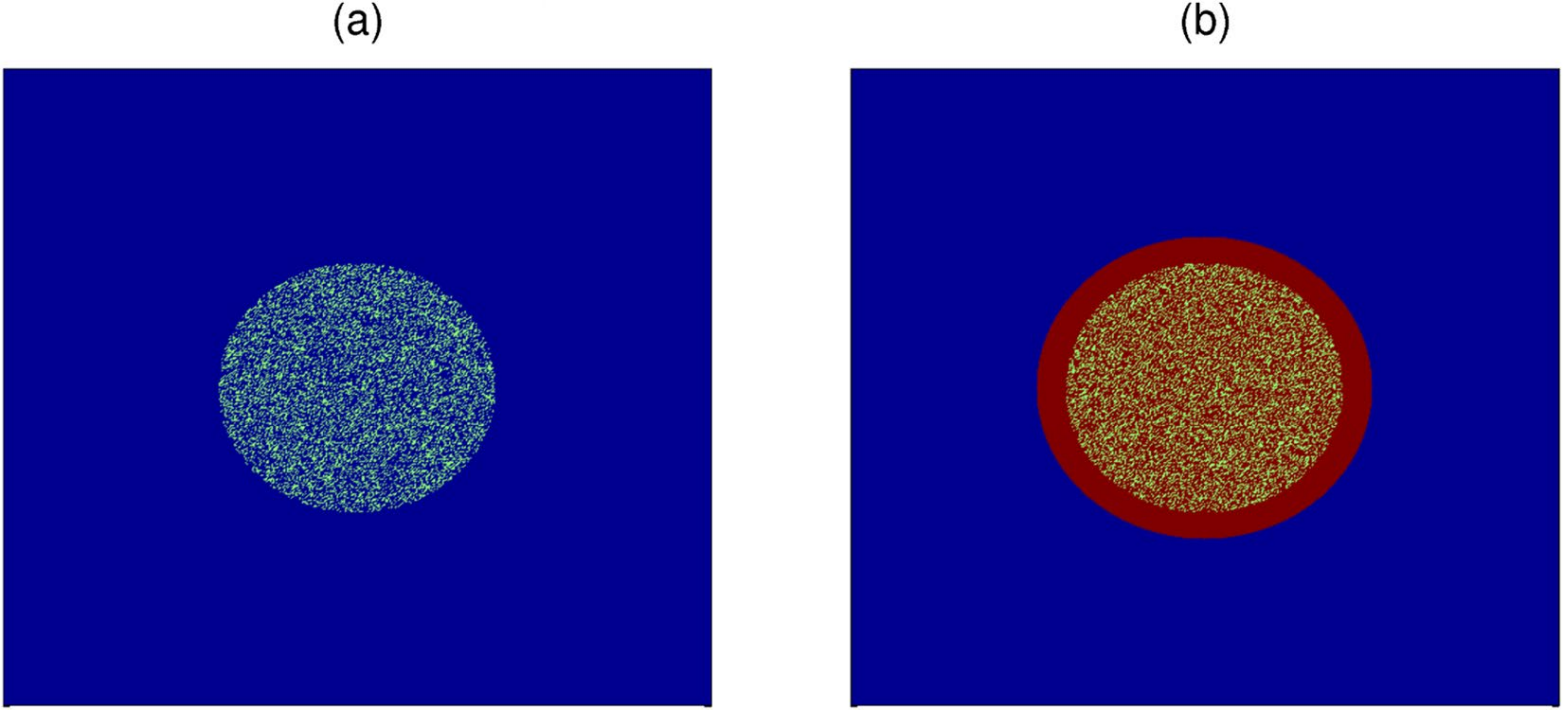
Fibroblast clumps. (**a**) Pseudocolour plot of a domain with a fibroblast clump; inside the circular clump fibroblasts are distributed randomly such that the percentage of sites with fibroblasts is ${p}_{f}$ = 30%. In our model, a site can be occupied either by a myocyte or by a fibroblast, but not both; blue and yellowish-green colours indicate normal myocytes and fibroblasts, respectively. (**b**) A fibroblast clump, as in (**a**), where the excitability of the myocytes, inside and around the clump, is modified. The red colour indicates myocytes with modified excitability (see text).

**Figure 2:**
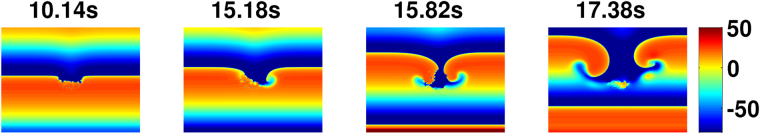
Wave distortions and spiral waves, around a fibroblast clump, caused by high-frequency pacing (PP stimulation protocol). Pseudocolour plots of ${{\rm{V}}}_{{\rm{m}}}$ showing the formation of wave distortions, because of high-frequency pacing and with the protocol PP (see text), at a fibroblast clump; in this figure we use the illustrative parameters ${p}_{f}\mathrm{=30}$% and radius R = 1 cm. Note that WD occurs first at the periphery of the fibroblast clump at time t = 15.18 s; eventually this WD evolves into spiral waves at t = 17.38 s.

We show that a localized distribution of fibroblasts can induce spiral waves if we pace the medium at a high frequency. We consider a circular clump of radius R = 1 cm, with a random distribution of fibroblasts inside it, as shown in Fig. 1(a) for the illustrative case ${p}_{f}=\mathrm{30 \% }$. We use the PP protocol, with a pacing cycle length (PCL) of 152 ms (i.e, a pacing frequency = 6.6 Hz, which is of the order of the spiral-wave frequency $\omega$ $\simeq$ 6.4 Hz). We use this value of PCL value for all the high-frequency-pacing studies in the remaining part of this paper. At this high-frequency pacing, as the waves interact both with the surface and the inside of the fibroblast clump, they get distorted. Prominent distortions of the waves occur near the surface of the clump (see Fig. 2); and these distortions grow in time and lead to the formation of spiral waves, as shown in Fig. 2 (see the Video S1 in the Supplementary Material for the complete spatiotemporal evolution).

**Figure 3:**
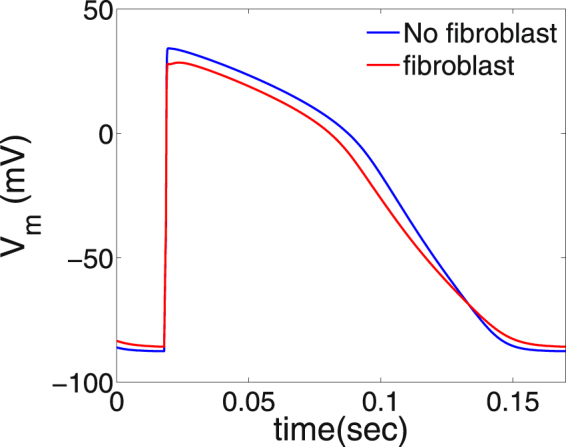
Action potentials. Figure showing the action potentials of an isolated myocyte (blue curve) and a myocyte coupled to a fibroblast (red curve). We see that the APD of the red-curve is slightly larger than the APD of the blue curve, which indicates that coupling of a myocyte to a fibroblast slightly increases the APD of the myocyte.

The reason for the occurrence of wave distortions (WD) near the surface of the clump is as follows. The coupling of fibroblasts with the myocytes increases slightly the action potential duration (APD) of the myocytes, because fibroblasts in our model have a higher resting membrane potential (−30 mV) than myocytes (−90 mV). Figure 3 shows the action potentials of an isolated myocyte (blue curve) and a myocyte coupled to a fibroblast (red curve). We see a slight increase in the APD of the myocyte coupled to a fibroblast, which leads to a small increase of the effective refractory period (ERP) of the region inside and around the fibroblast clump. Moreover, given the random distribution of fibroblasts inside the clump, the waves travel along serpentine paths; this reduces the effective velocity of the waves inside the clump^28,43,50^. Because of the low velocity of the wave inside the clump, the medium in the clump gets activated later, and, hence, also gets repolarized later, than the medium outside the clump. Therefore, because of this late repolarization and a slight increase of ERP of the medium in the clump, as the succeeding wave propagates in the clump it encounters a medium which is not completely repolarized. This interaction of the succeeding wave with an unrepolarized medium, after the passage of a preceding wave, is called the waveback-wavefront interaction. Given that (a) the surface of the clump is an interface between the low-wave-velocity region, inside the clump, and the high-wave-velocity region, outside the clump, (b) the ERP of the region around the fibroblast clump is slightly higher than that of the region far away from the clump, and (c) there is a random distribution of fibroblasts, the waveback-wavefront interactions at the surface of the clump induces corrugations on the wavefronts of the waves. The corrugations on the wavefront of the propagating waves in a medium with fibroblasts is a well-known phenomenon^28,43,51^. These corrugations on the wavefronts, near the surface of the clump, becomes develop with time, as we pace the medium, and lead, eventually, to the formation of wave distortions that initiate the spiral waves. The occurrence of this WD relies on the waveback-wavefront interactions between the preceding and succeeding waves; and, therefore, such distortions occur much more readily with high-frequency pacing than with low-frequency pacing; e.g., if we increase the PCL to 200 ms (i.e, a pacing frequency =5 Hz, which is lower than the spiral-wave frequency $\omega$ $\simeq$ 6.4 Hz), we find no WD, and, hence, no spiral wave is formed in the medium (see Video S2 in the Supplementary Material). If we use low-frequency (i.e., large-PCL) pacing, after the passage of a wave, the region around the clump gets enough time to recover before the arrival of the succeeding wave; therefore, there is no waveback-wavefront interaction between the consecutive waves; consequently, we do not see any WDs when we use low-frequency pacing. This occurrence of spiral waves via high-frequency pacing in a medium with a structural heterogeneity has also been demonstrated in the case of a medium with sharp-edged obstacles^52^. We also check our simulation with a value of $\delta x=0.006$ cm, which is smaller than the default value of 0.02 cm, just to ensure that the formation of WDs is not because of a large spatial resolution in the dicretization of our domain. We find that the formation of WDs still occur in this case (Fig. S2 in the Supplementary Material) with the 0.006 cm spatial resolution.

**Figure 4:**
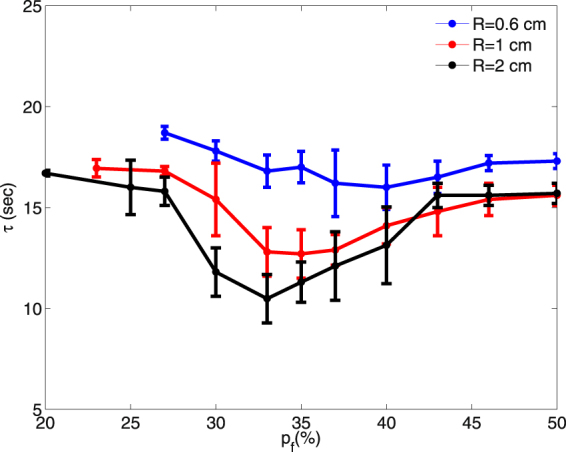
Plots of $\tau$, the mean time for the formation of wave distortions (WDs), versus ${p}_{f}$. The variation of $\tau$ with the percentage ${p}_{f}$ of fibroblasts, in the clump, for clump radii R = 0.6 cm (blue curve), 1 cm (red curve), and 2 cm (black curve); we obtain errors bars from the standard deviation of $\tau ^{\prime}$ (see text). $\tau$ decreases, initially, with an increase of ${p}_{f}$; but then it increases with ${p}_{f}$ and eventually saturates. For fixed ${p}_{f}$, the larger the value of R the smaller is the value $\tau$.

We see that the formation of spiral waves in the medium is preceded by the WD around the fibroblast clump; therefore, it is important to study the factors that affect the initiation of such WDs. We find that the time $\tau ^{\prime}$, at which WD occurs, depends *on the precise realisation of the random configuration of fibroblasts*. Therefore, for values of ${p}_{f}$, which lead to WDs, we calculate the average value of $\tau ^{\prime}$, which we denote by $\tau$, over 8 realizations of the fibroblast distribution for each one of the values of R that we consider. In Fig. 4 we show how $\tau$ varies with ${p}_{f}$ for R = 0.6 cm (blue curve), 1 cm (red curve), and 2 cm (black curve). Let us examine the dependence of $\tau$ on ${p}_{f}$ for the illustrative case of the fibroblast clump with R = 1 cm (red curve): Below a threshold value ${p}_{f}\simeq$ 23% (and this threshold value of ${p}_{f}$ depends on the clump size R), we do not observe any WD given our simulation time. We only observe WD for ${p}_{f}\ge$ 23% after a certain period with $\tau$ = 16.8 ms for R = 1 cm. The value of $\tau$ decreases with an increase of ${p}_{f}$ until some ${p}_{f}$ value (35% for R = 1 cm); thereafter, $\tau$ increases with ${p}_{f}$ and, finally, saturates at a value that depends on R. The variation of $\tau$ with ${p}_{f}$ can be explained as follows. At very low ${p}_{f}$ ($\le$23%, for R = 1 cm) the number of fibroblasts is too low to induce significant heterogeneneity in the peripheral region of the clump, and, hence, we do not see any distortions of the waves at low ${p}_{f}$; however, beyond the threshold value ${p}_{f}=$ 23%, the fibroblasts begin to disrupt wave propagation in the region around the fibroblast clump; this leads to WDs, and, eventually, spiral waves. In the intermediate range 23% $\le \,{p}_{f}\mathop{ < }\limits_{ \tilde {}}$ 37%, the periphery of the fibroblast clump becomes more and more jagged; this favours the formation of WDs, so $\tau$ decreases as ${p}_{f}$ increases in this range. At higher values of ${p}_{f}$, the clump becomes more and more impenetrable because of the high density of fibroblasts; moreover, as ${p}_{f}$ increases beyond 37%, the periphery of the clump becomes smooth, because more and more sites in the peripheral region are now occupied by fibroblasts; consequently the formation of WDs is somewhat suppressed and $\tau$ increases slightly as ${p}_{f}$ increases and then saturates at large values of ${p}_{f}$.

Another factor that controls the occurrence of WD is the size of the fibroblast clump. For fixed ${p}_{f}$, the larger the value of R the smaller is the value $\tau$ (Fig. 4), because the larger the clump, the longer is its jagged periphery with which the wave interacts. Hence, WDs occur more readily with large fibroblast clumps than with small ones.

Although we have used a random distribution of fibroblasts in the clump that leads to a uniform mean density, the formation of WDs should hold even in the case of a clump with a random distribution of fibroblasts that leads to a mean density which is not uniform in space. We show an illustrative case in Fig. S3 in the Supplementary Material, where the mean density of fibroblasts decreases linearly from ${p}_{f}$ = 35%, in the central region of the clump, to ${p}_{f}$ = 0% at the periphery of the clump. We see that WDs still occur for the case of a clump with such a distribution of fibroblasts.

Moreover, there is some evidence for fibroblasts being active cells^42,53^. Therefore, we carry out a representative simulation by using the active-fibroblast model due to MacCanell, *et al*.,^42^. We still find that WDs occur at high-frequency pacing, as shown in Supplementary Fig. S4. This is expected because the main reason of the formation of WDs is the structural heterogeneity induced by the fibroblasts; and, therefore, WDs can occur so long as the presence of fibroblasts induces gap-junctional interruptions between the myocyte couplings.

### Effects of electrical remodelling of the tissue near a fibrotic clump

Diseases like ischemia or myocardial infarction, which trigger fibrosis, can also cause electrical remodelling of the tissue near a fibrotic clump;^32,54^ such ionic remodelling can change the excitability of the medium in the vicinity of the clump. Therefore, we study the effects on WDs of changes in the excitability of the medium, inside and regions around the fibroblast clump.

**Figure 5:**
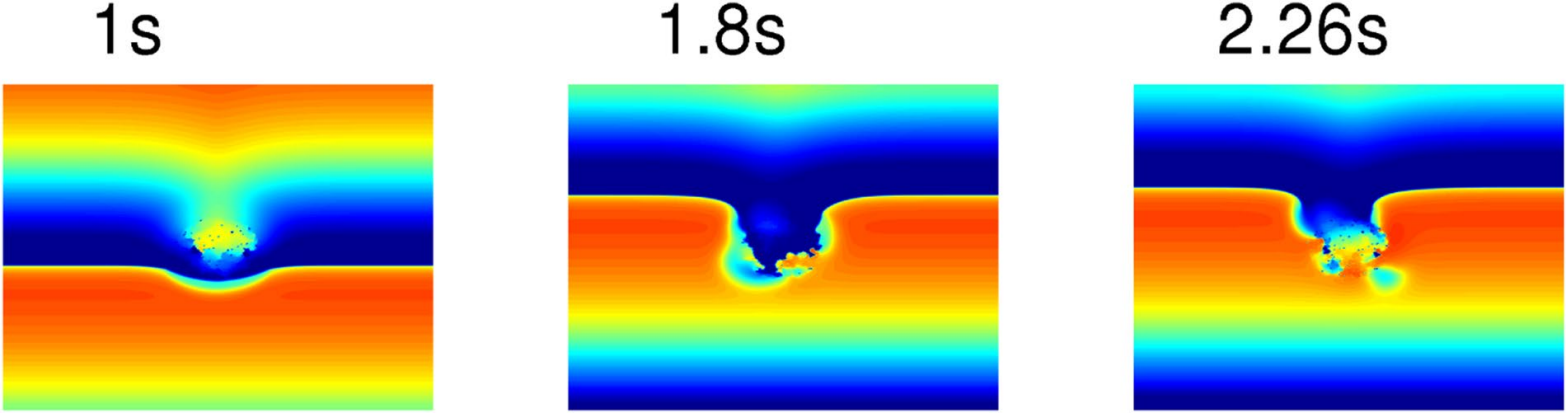
Early occurrence of WD on the reduction of excitability inside and around a fibroblast clump. Pseudocolour plot of ${V}_{m}$ showing the early occurrence of WD at t $\simeq$ 1.8 s for the illustrative case of a fibroblast clump with R = 1 cm and ${p}_{f}$ = 30%. Here, we reduce the excitability inside the clump and in an annular region around it (marked by red colour) within a circular region with radius of 1.4 cm (Fig. 1(b)), by reducing the conductance of ${{\rm{I}}}_{{\rm{Na}}}$ (${{\rm{G}}}_{{\rm{Na}}}$) by 50%.

Let us first consider the effects of a reduction of this excitability for the illustrative case of a fibroblast clump with R = 1 cm and ${p}_{f}$ = 30%. We reduce the excitability inside the clump and in an annular region around it, i.e., in the red-coloured circle with a radius of 1.4 cm (Fig. 1(b)) by reducing the conductance of ${{\rm{I}}}_{{\rm{Na}}}$ (${{\rm{G}}}_{{\rm{Na}}}$) by 50%; such reduction in ${{\rm{G}}}_{{\rm{Na}}}$ is observed in infarcted regions of cardiac tissue^49^. This reduction in the excitability of the region around the clump leads to WDs much earlier than with normal excitability, i.e., with the control value of ${{\rm{G}}}_{{\rm{Na}}}$. Figure 5 shows the occurrence of WD roughly after t = 1.8 s (for a particular realization of the random distribution of fibroblasts), when the excitability is reduced in the region inside and around the clump. The value of $\tau$ with this reduced excitability is $\simeq$1.7 s, which is considerably lower than τ ≃ 15.4 s (see Fig. 4) for the case with normal excitability. This reduction of $\tau$ is a consequence of the slowing down of the wave in the region with low excitability, inside and around the clump; the slow propagation of waves here allows for more prominent waveback-wavefront interactions in the peripheral region of the clump, and, therefore, an easier initiation of WD than in the case with normal excitability.

Myocardial infarction, that cause fibrosis, is associated with changes in the properties of many other ion channels^46,47^ apart from the sodium channel, and also modifications in gap-junctional conductances are observed in infarcted regions^48^. Therefore, we also carry out a representative simulation of a fibrotic tissue in which we consider remodelling of gap-junctional coupling and other ion-channels (${{\rm{I}}}_{{\rm{Kr}}}$, ${{\rm{I}}}_{{\rm{Ks}}}$, and ${{\rm{I}}}_{{\rm{CaL}}}$) in the region inside the fibroblast clump (fibrotic region) and also in the border zone, which separates the fibrotic region from the normal region. The details of the electrical remodelling of the fibrotic tissue are given in the Supplementary Material. We show, in Fig. S5 of the Supplementary Material, that, even with this remodelling, the early occurrence of the wave distortions still holds.

**Figure 6:**
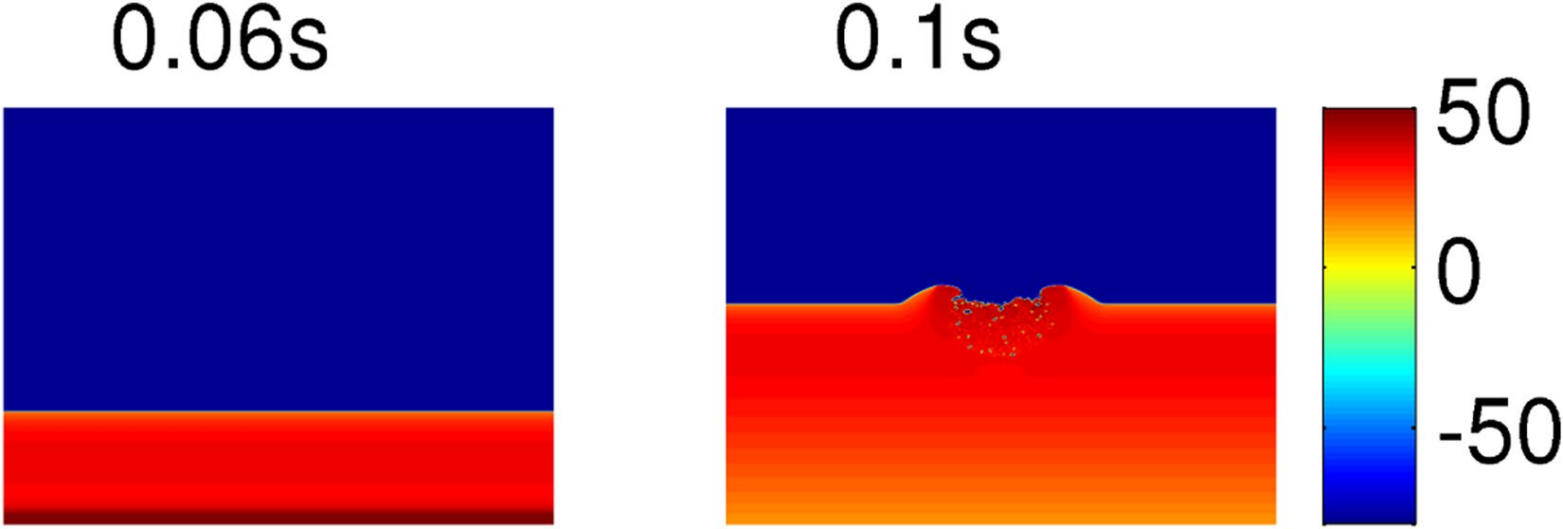
Increase of the wave velocity around the fibroblast clump with enhanced excitability in and around the clump. Pseudocolour plot of ${V}_{m}$ showing the increase in the wave velocity in the vicinity of the fibroblast clump with ${p}_{f}$ = 30%, R = 1 cm when the excitability of the medium is increased (${G}_{Na}$ $\times$ 1.1). The bump on the wavefront at t = 0.1 s occurs because the wave propagates fast in the vicinity of the clump.

We also study how an increase in the excitability of the medium affects the formation of WDs. To carry out this study, we increase ${{\rm{G}}}_{{\rm{Na}}}$ by 1.1 times its original value in the region inside and around the clump (Fig. 1(b)) and, thereby, the propagation velocity of the waves in this region, as we show in Fig. 6. The bump in the wavefront of the plane wave in the region near the clump in Fig. 6 (t = 0.1 s) occurs because the wave propagates faster in that region than in the rest of the medium. The faster a wave propagates in the region near the clump, the earlier is the activation of the tissue here, and, therefore, the earlier is recovery of the tissue in that region. This early recovery of the tissue in the region near the clump reduces the waveback-wavefront interaction and, therefore, disfavours the formation of WDs. For the illustrative case of a clump with ${p}_{f}$ = 30%, R = 1 cm, and ${G}_{Na}$ 1.1 times its original value, we find that $\tau$ increases to $\simeq$18 s, which is larger than its value with normal excitability ($\tau$ = 15.4 s). The Supplementary Video S3 shows, for one of our realizations of a clump with ${p}_{f}$ = 30%, R = 1 cm, that WD occurs at t $\simeq$ 17.7 s. If we increase the excitability considerably, say by increasing ${{\rm{G}}}_{{\rm{Na}}}$ to 3 times its original value, inside and around the fibroblast clump, then no WDs occur over the duration of our simulations. This implies that an increase in the excitability of the myocytes in such a region prevents the occurrence of WD, and, hence, inhibits the formation of spiral waves that can occur because of high-frequency pacing.

### Reentrant waves in a fibrotic region

#### 2D simulation

**Figure 7:**
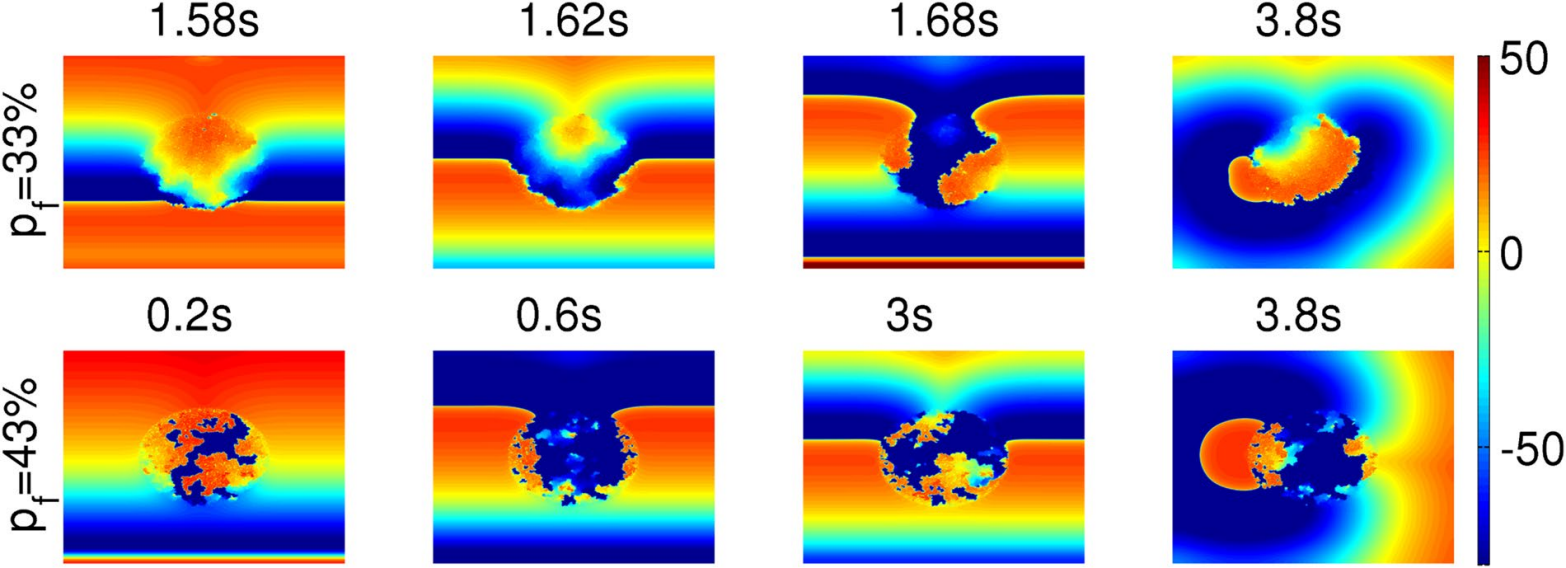
Reentry via the high-frequency TP protocol. Pseudocolour plot of ${V}_{m}$ showing the initiation of reentry, because of our high-frequency pacing (TP protocol), in a medium with a fibroblast clump with R = 2.4 cm and ${p}_{f}$ = 33% (top row) and 43% (bottom row).

The random distribution of fibroblasts creates tortuous channels, for the waves that enter the clump, and, thereby, pathways for reentry inside the clump. Here, we show how reentry can occur in the medium through a different process, which does not involve the formation of WDs like in Fig. 2, by using the following transient-pacing (TP) protocol: we apply 20 rapid pulses and cease pulsing after that. We illustrate this TP-induced reentry for a fibroblast clump with R = 2.4 cm and ${p}_{f}$ = 33% in Fig. 7 (top row). This reentry can be understood qualitatively by considering the clump-mediated waveback-wavefront interactions. The waves, as discussed earlier, travels more slowly through the clump than through the region outside it. Therefore, as the first wave propagates through the medium the region inside the clump gets excited later than the region outside the clump. This difference in the activation time between the inside and outside regions leads to delayed recovery inside the clump; consequently the waveback is uneven (see Fig. 7 top row at t = 1.58 s). Therefore, if we use high-frequency pacing (PCL = 152 ms), after a wave passes through the clump, the succeeding wave encounters, on its arrival, a refractory region inside the clump. This leads the succeeding wave to propagate around the clump (see Fig. 7 top row t = 1.62 s); and it finally re-enters the region inside the clump (see Fig. 7 t = 1.68 s) once the region recovers and, thus, leads to reentry in the medium. At high values of ${p}_{f}$, say ${p}_{f}$ = 43% (Fig. 7, bottom row), we also see fractionation of waves inside the clump because of the presence of high density of fibroblasts (see also the Supplementary Video S4).

**Figure 8:**
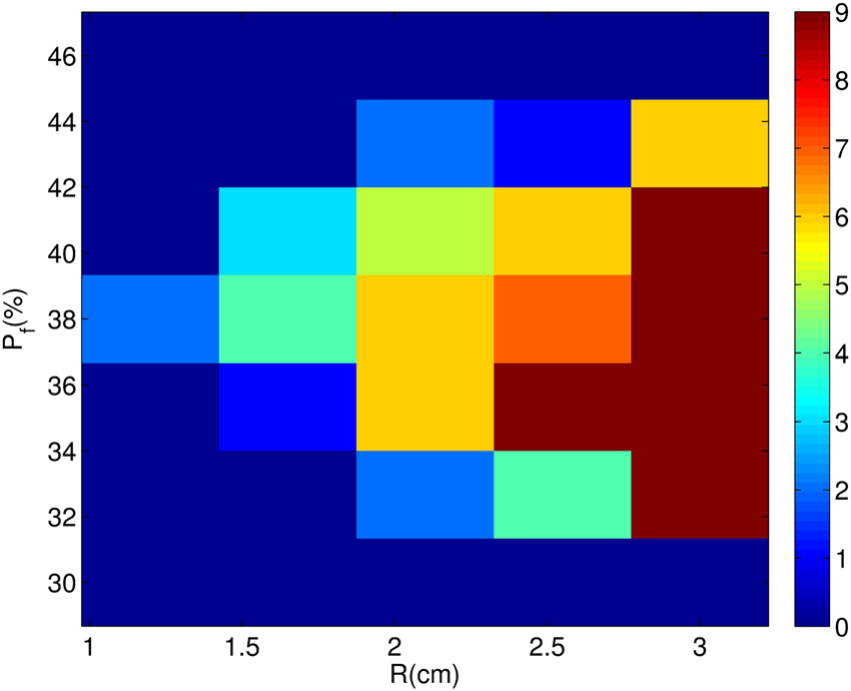
Stability diagram, showing regions in which reentry occurs in the R-${p}_{f}$ plane when we stimulate a medium with a clump of fibroblasts by using the high-frequency TP stimulation protocol. We find no reentry in the outermost, dark-blue region of the R-${p}_{f}$ plane. The different colours indicate the number of times we obtain reentry in the 10 simulations (for each pair of values of ${p}_{f}$ and R, we perform 10 simulations for 10 different realizations of the fibroblast distribution inside the clump). We use the TP-pacing protocol (see text) with PCL = 152 ms. The colours indicate the total number of reentries we obtain in our simulations for 10 different realizations of the random distribution of fibroblasts.

Such TP-induced reentrance depends on R, the linear size of the clump, the density $${p}_{f}$$ of fibroblasts, and also *the realization of the random distribution of fibroblasts*. In Fig. 8, we find no reentry in the outermost, dark-blue region of the R-$${p}_{f}$$ plane. For each pair of values of $${p}_{f}$$ and R, we perform 10 simulations for 10 different realizations of the fibroblast distribution inside the clump; reentry occurs for some of these realizations and not for others. The different colours in Fig. 8 indicate the number of times we obtain reentry in the 10 simulations for a given set of values for $${p}_{f}$$ and R. We can deduce from Fig. 8 that, for a given value of $${p}_{f}$$, reentry occurs more often for large R than for small R. Also, we see that reentry is most prominent at intermediate values of $${p}_{f}$$, which we explain as follows: at very low values of $${p}_{f}$$ ($${p}_{f} < $$ 30%), the number of fibroblasts is too low to induce significantly uneven wave propagation in the medium; and, at very large values of $${p}_{f}$$ ($${p}_{f} > $$ 46%), the clump becomes impenetrable, because of the high density of fibroblasts and, hence, provides no path for reentry.

**Figure 9:**
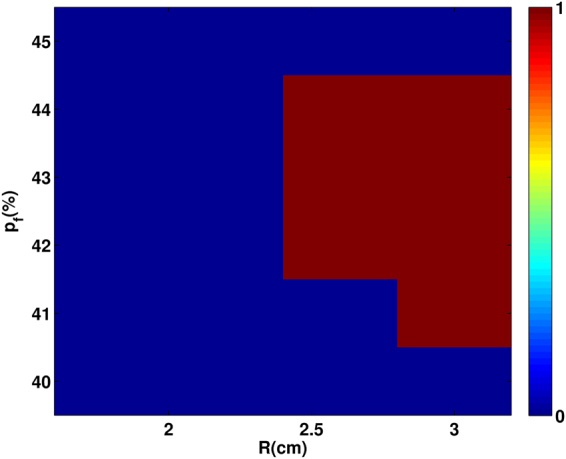
Region in the R-${p}_{f}$ plane in which reentry occurs via our low-frequency TP stimulation protocol. A stability diagram showing regions, in the R-${p}_{f}$ plane, where reentry occurs (red) and where it does not (blue), with our TP-pacing protocol for PCL = 200 ms. The colours also indicate the total number of reentries we observe in our simulations for 10 different realizations of the random distribution of fibroblasts.

Let us now consider TP-induced reentry at low pacing frequencies, i.e., large PCL; we use PCL = 200 ms here. We find that reentry reduces drastically relative to the case of high-frequency pacing as we can see by comparing the R-${p}_{f}$ plane stability diagram of Fig. 9 with its high-frequency counterpart Fig. 8.

The reason for this reduction is as follows: At low-frequency pacing, as the time separation between the arrival times of two consecutive waves is large, the region inside the clump gets enough time to recover, almost fully, before the next wave advances through it, so the wavefront-waveback interaction is attenuated and, in turn, reduces the chances of reentry.

**Figure 10:**
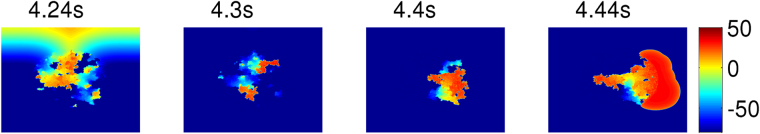
Reentry via our low-frequency TP stimulation protocol. Pseudocolour plot of ${V}_{m}$ showing the formation of reentry, via our low-frequency TP pacing protocol, in a medium with a fibroblast clump of size R = 3 cm and ${p}_{f}$ = 41%. After the passage of the last (20th) wave, the irregular wave pattern (t = 4.3 s) left inside the clump triggers excitations that leads to reentry (see text).

In particular, for our low-frequency TP-protocol study, we find reentry only in the narrow range 41% $\le$ ${p}_{f}$ $\le$ 44% and with R $>$ 2.4 cm (see Fig. 9); this region is shifted to higher values of ${p}_{f}$ relative to its counterpart in the high-frequency case (Fig. 8). In this ${p}_{f}$ range of 41–44%, the occurrence of reentrant phenomenon is because of the fractionation of waves inside the clump. We show, in Fig. 10, the occurrence of reentry, with this low-frequency TP-protocol, for the illustrative case of a clump with ${p}_{f}$ = 41% and R = 3 cm. At such high values of ${p}_{f}$, the passage of waves through the clump leaves behind an irregular pattern of waves of excitation inside the clump (see Fig. 10 t = 4.24 s); these fractionated waves then trigger abnormal excitations that lead to reentry in Fig. 10. Once this reentry occurs, the fibroblast clump continues to emit waves and acts, therefore, like an ectopic focus. This phenomenon is similar to what has been reported in^33,34^ for a simple model, with a single-pulse protocol, which can be thought of as an extremely low-frequency-version of our TP protocol.

#### 3D simulation

**Figure 11:**
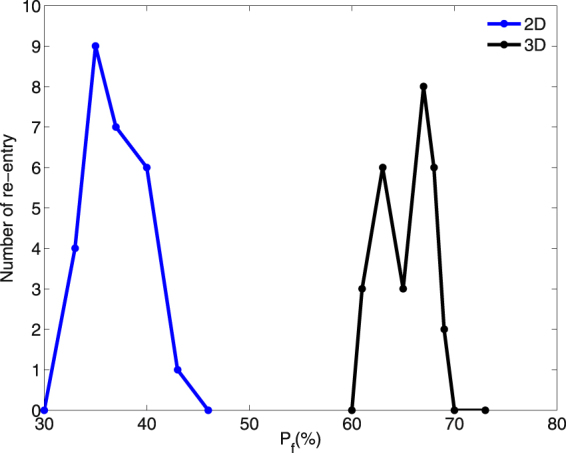
TP-pacing induced reentry in our 2D and 3D simulation domains with fibroblast clumps of R = 2.4 cm. Figure showing the range of ${p}_{f}$ (horizontal axis) in which reentry occurs, via our TP-pacing protocol with PCL = 152 ms, for 2D (blue curves) and 3D (black curves) simulation domains with fibroblast clumps of R = 2.4 cm (in the 3D case the clump is cylindrical). The vertical axis shows the total number of reentry episodes that we observe in our simulations for 10 different realizations of the fibroblast distribution.

**Figure 12:**
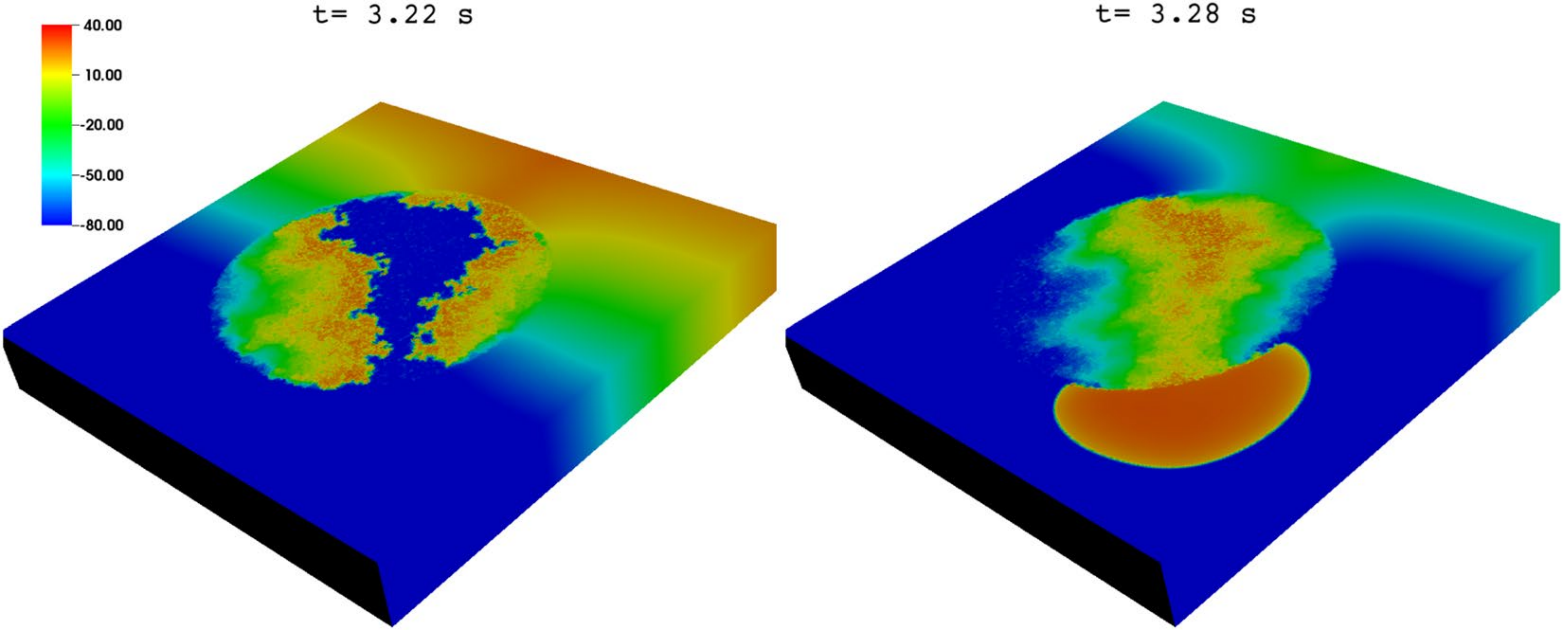
TP-pacing induced reentry in our 3D simulations with a cylindrical fibroblast clump. Pseudocolour plot of ${V}_{m}$ showing the formation of reentry, via our TP stimulation protocol with PCL = 152 ms, in a 3D domain with a cylindrical fibroblast clump, ${p}_{f}$ = 65%, and R = 2.4 cm.

We also study the occurrence of reentry, for the illustrative case of the TP stimulation protocol with PCL = 152 ms, in a three-dimensional (3D) simulation domain with a cylindrical fibroblast clump that has a radius R = 2.4 cm. We find that reentry occurs at higher value of ${p}_{f}$ in this 3D case as compared to that in our 2D study above. Figure 11 shows the values of ${p}_{f}$ for which reentry occurs in our 2D and 3D simulations for R = 2.4 cm. The values on the vertical axis denote the total number of reentry episodes that we observe in our simulations for 10 different realizations of the fibroblast distribution; and the horizontal axis displays the values of ${p}_{f}$. The blue and black coloured curves are from our 2D and 3D simulations, respectively. Figure 11 shows that reentry occurs in the 2D case in the range 33% $< {p}_{f} <$ 43%, whereas, in the 3D case, it occurs in the range 63% $< {p}_{f} <$ 68%. These ranges straddle the site-percolation thresholds for square and simple-cubic lattices^33,34^; note that our variable ${p}_{f}$ is, roughly speaking, $\mathrm{(1}-p)$, where $p$ is the probability that a site is occupied in the conventional site-percolation problem. Such a shift in the range of ${p}_{f}$ for reentry in 3D domain as compared to that in a 2D domain was also observed in the study of Alonso, *et al*.^34^, with their single-pulse protocol. The reason behind the shift in this range, as we increase the dimension (with the value of R held fixed), is as follows: The nearest-neighbour coordination number is higher in our 3D, simple-cubic-lattice domain than in our 2D, square-lattice domain. Therefore, for a given value of ${p}_{f}$, the average number of neighboring myocytes of a mycoyte cell is higher in 3D than in 2D, so a wave propagates more easily in the 3D case than in the 2D case^28^: Disorder induced by fibroblasts (in the myocyte gap-junctional coupling) is much more disruptive in 2D than in 3D. Figure 12 shows the occurrence of reentry in our 3D simulation with a fibroblast clump of R = 2.4 cm and ${p}_{f}$ = 65% (see also the Supplementary Video S5).

## Discussion

We have investigated how a cluster of fibroblast cells, distributed randomly inside a region that we call a clump, can induce reentry in the medium when we apply periodic pulses. By using our persistent pacing (PP) protocol, we have first shown that fibroblast clumps can induce spiral waves, at high-frequency pacing, because of interactions between the wavebacks and the wavefronts of consecutive waves. We find that after a wave propagates through the fibroblast clump it leaves behind a heterogeneous region around the clump, which distorts the wavefront of a succeeding wave in the peripheral region of the clump. This wave distortion (WD) effect is transferred to the succeeding waves, and the distortion grows in time and leads, eventually, to the initiation of spiral waves (see Fig. 2). For low-frequency pacing, the time separation between consecutive waves is large, so the region around the clump recovers before the arrival of the next wave and does not induce significant waveback-wavefront interactions. Therefore, we see no WDs and, hence, no spiral waves at low-frequency pacing. The average time $\tau$, after which WD occurs, depends on ${p}_{f}$ and the size R of the fibroblast clump as we show in Fig. 4. Moreover, we find (see Fig. 5) that WDs occur early (i.e., we have small values of $\tau$) if we reduce the excitability (e.g., by decreasing the value of ${G}_{Na}$) inside and around the clump; an increase in the excitability has the opposite effect, i.e, it delays the formation of WDs. This is an important finding that may be of clinical relevance, because the occurrence of fibrotic tissue is known to be associated with electrical remodelling of cardiac tissue^54,55^.

We also find that reentry can occur through a different process, which does not involve the intermediate step of the formation of WDs. In particular, by using our transient-pacing (TP) protocol, we have shown that waves can reenter the medium through the fibroblast clump (see Fig. 10). Here reentry occurs because the region inside the clump recovers later than the region outside it, as a result of the slower propagation of waves inside the clump than in the region outside it. Hence, a waveback-wavefront interaction is induced by the fibroblast clump; this leads to reentry. The waveback-wavefront interaction is more prominent at high-frequency pacing than at low-frequency pacing, so reentry occurs more readily in the high-frequency case than in the low-frequency one (compare Figs 8 and 9). Finally we show that the range of ${p}_{f}$, over which reentry occurs, via our TP-stimulation protocol, lies at higher values of ${p}_{f}$ for our 3D simulation domain than in our 2D domain.

Many earlier studies have investigated the arrhythmogenic effects of fibroblasts. In particular, it has been shown that fibroblasts, distributed randomly in a monolayer with myocytes, can cause fragmentation of waves^25,43^ and even complete waveblock^25^. The presence of local^26^ and global^27^ gradients in the density of fibroblasts has also been shown to induce spiral waves. Other studies have investigated the effects of fibroblast on the stability of spiral waves^28–31^. Apart from the structural heterogeneity that is imposed by fibroblasts to a wave in a monolayer of myocytes, the coupling of fibroblasts with myocytes is also known to promote pathological excitations like alternans^56^ and early afterdepolarizatins (EADs)^57,58^.

Most of the studies mentioned above investigate the effects of fibroblasts on wave dynamics where the fibroblasts are distributed throughout the domain. However, in diseased hearts, fibrosis often leads to regional occurrences of fibroblasts^32,59^, which do not proliferate all over the tissue. Such localized distributions of fibroblasts can occur, e.g., after an incidence of myocardial infarction^32^. Therefore, it is important to study the effects of the coexistence of such fibrotic regions with normal region in cardiac tissue. Some studies have investigated the arrhythmogenic effects of localized fibroblast distribution in the context of the triggering of abnormal excitations like EADs^57,58^. Moreover, studies with more realistic representation of localized fibrotic tissue, in particulra, those with fibrosis patterns derived from patients, have been performed^60–63^. Our study, which illustrates the two processes of spiral-wave initiation via PP and TP pacing protocols, enriches the understanding of the formation of reentry in such fibrotic hearts. The studies by Alonso, *et al*.^33,34^, have found that, in a medium with a clump of fibroblasts, reentry occurs most often near the percolation threshold; however, they have not investigated the reentry-initiating potential of fibroblast clumps when the medium is paced. Our results show clearly how such pacing has a strong influence on the initiation of reentry by such fibroblast clumps. For example, spiral-wave initiation via WDs (see Fig. 2) only occurs at high-frequency pacing and not at low-frequency pacing; and, with our TP protocol, reentry occurs more readily with high-frequency rather than low-frequency pacing, i.e., a fibroblast clump is much more arrhythmogenic if the pacing frequency is high than if it is low.

We end our discussion with some limitations of our work. In our 2D- and 3D-tissue calculations we do not take into account the tissue anisotropy that can arise from the orientations of muscle fibres in real heart tissue; and fibrosis is known to change the local fiber orientations in and around the fibrotic regions^64^. Such changes in fiber orientations can affect the dynamics of waves^65^. The extension of our work to tissue models that consider fiber architechture must await future studies. We have used a monodomain representation of cardiac tissue; our study needs to be extended to other tissue models like bidomain models^66^. Furthermore, our study, like many other computational studies^29–32^, considers a myocyte-fibroblast (MF) coupling; however, such coupling has not been observed directly in *in-vivo* experiments so far, although it has been demonstrated in *in-vitro* experiments^35–37^. Our study is conducted by using a passive model (in all but once case) for the fibroblast cells in our simulations; the qualitative results of our study do not depend on the fibroblast models, as we illustrate in Supplementary Fig. S5 the formation of WDs in an active-fibroblast model.

## Electronic supplementary material

Video S1

Video S2

Video S3

Video S4

Video S5

Supplementary Material
