# Supplementary material for: Reentry via high-frequency pacing in a mathematical model for human-ventricular cardiac tissue with a localized fibrotic region
Source: Sci Rep. 2017 Nov 10;7:15350. doi: 10.1038/s41598-017-15735-5 (PMC5681702; doi:10.1038/s41598-017-15735-5)
Supplement: Supplementary file 6 — Supplementary Material [file 41598_2017_15735_MOESM6_ESM.pdf]

Supplementary Material

Reentry via high-frequency pacing in a mathematical model for human-ventricular cardiac tissue with a localized fibrotic region

Soling Zimik[1] and Rahul Pandit[1]

[1]Centre for Condensed Matter Theory, Department of Physics, Indian Institute of Science, Bangalore, 560012, India

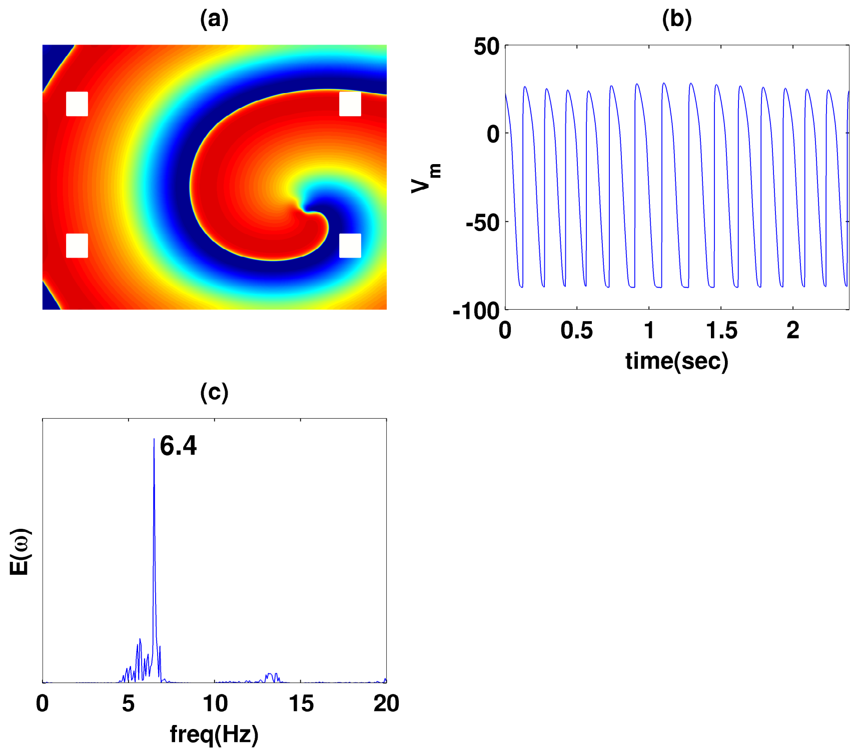

Figure S1: Spiral wave. (a) Pseudocolour plot of V_m illustrating a spiral wave. The white squares indicate the points from where we record the time series of V_m. (b) Time series of V_m from one of these representative points. (c) The averaged power spectrum of these time series; this is the average of the power spectra of the time series from the four representative points mentioned above. The spiral-wave frequency ω is the value of the frequency of the dominant peak in this spectrum ,i.e, the peak at 6.4 Hz.

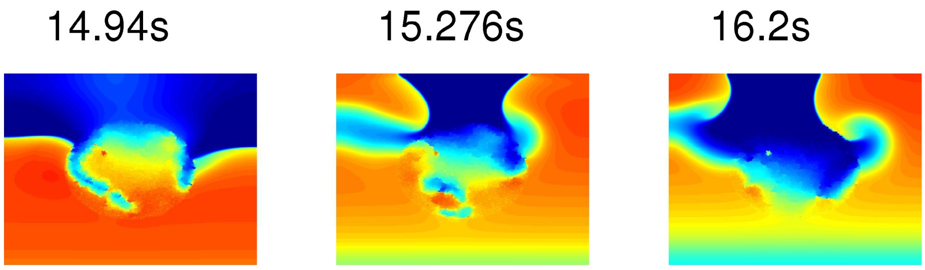

Figure S2: Wave distortion (WD). Pseudocolor plots of the transmembrane potential showing WD in a medium with a fibroblast clump of radius r=1 cm. The size of the domain is 640×640 grid points, with a spatial resolution δx= 0.006 cm and a temporal resolution δt=0.003 ms.

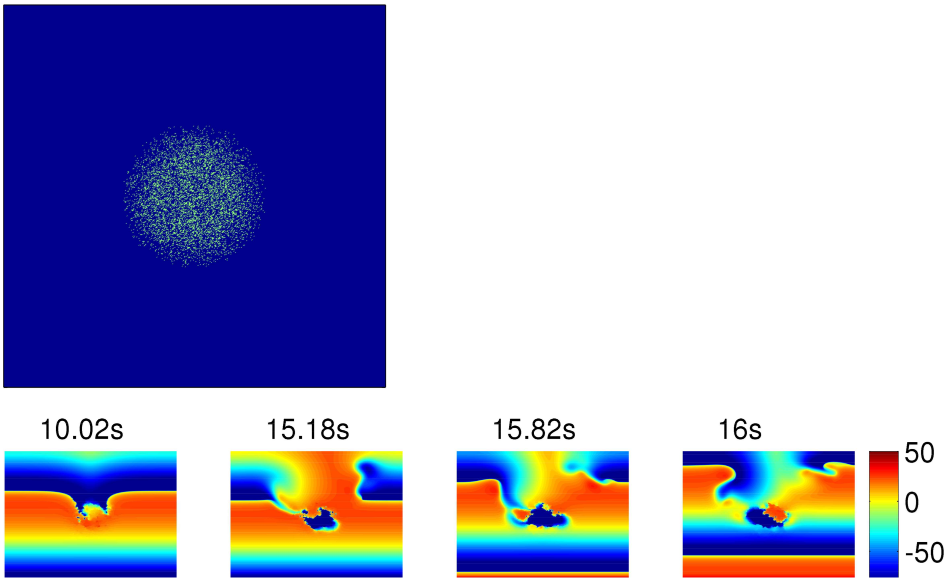

Figure S3: Wave distortion in a medium with fibroblast clump of with a random fibroblast distribution that leads to a nonuniform mean density. Top-panel: the distribution of fibroblasts in the clump. The percentage p_f of fibroblasts is 35%, within a radius of 1 cm; and then it decreases linearly to zero (in the normal region), within an annular region with width of 0.4 cm. Bottom-panels: pseudocolor plots of V_m showing the formation of WDs in the medium with this distribution of fibroblasts.

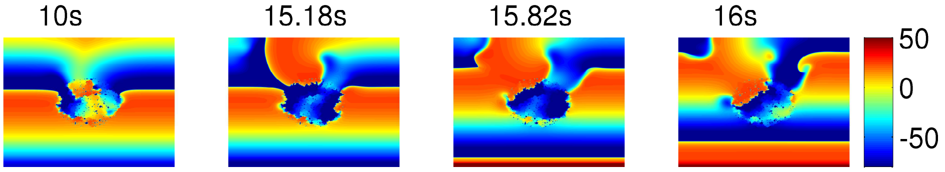

Figure S4: Wave distortion (WD) in an active-fibroblast model. Pseudocolour plots of V_m showing the formation of wave distortions around a fibroblast clump; here, we use the active-fibroblast model due to MacCanell, et al.,[42]

1 Model of fibroblast clump with remodelling in gap-junctional coupling and four ionic currents

We divide the medium into three regions (see fig. S5), namely, the normal region, the border zone (BZ), and the central zone (CZ); CZ lies inside the fibroblast clump; the BZ region is the region between CZ and the normal region. In the BZ region we reduce the conductances of I_{Na} by 50%, I_{CaL} by 50%, I_{Kr} and I_{Ks} by 30%. In the CZ region we reduce the conductances of I_{Na} by 70%, I_{CaL} by 70%, I_{Kr} and I_{Ks} by 60%. Furthermore, the value of the diffusion coupling is linearly reduced from its control value in the normal region to 60% of its original value in the CZ region.

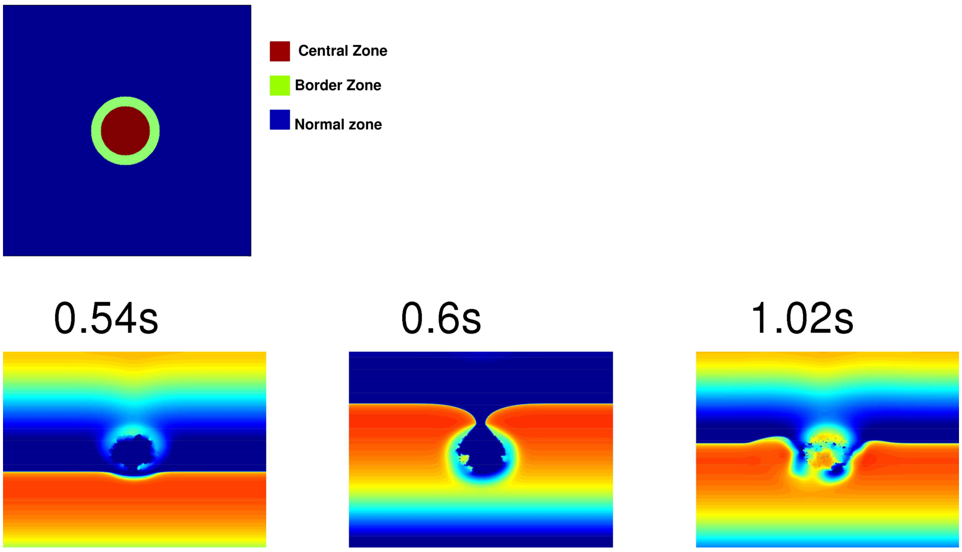

Figure S5: Wave distortion (WD) around a fibroblast clump of r= 1 cm and p_f=30% with electrophysiological remodelling. Top-panel: the normal region (blue), the BZ border zone (light green), and the CZ central zone (brown). Bottom-panel: pseudocolour plots of V_m showing the occurrence of WDs at t= 1.02 s (this is much earlier than the WD-initiation time τ=15.4 s without electrical remodelling).

Video captions

Video S1: **Wave distortions around a fibroblast clump via high-frequency PP protocol.** Video showing the formation of wave distortions and spiral waves around a fibroblast clump of p_f = 30% and radius R = 1 cm. For this video, we use 10 frames per second with each frame separated from the succeeding frame by 20ms in real time.

Video S2: **Absence of wave distortions around the fibroblast clump at low-frequency PP protocol.** Video showing that no wave distortions occur around a fibroblast clump of p_f = 30% and radius R = 1 cm if we use low-frequency PP protocol. For this video, we use 10 frames per second with each frame separated from the succeeding frame by 20ms in real time.

Video S3: **Delayed occurrence of wave distortions around the fibroblast clump with higher excitability.** Video showing that the formation of wave distortions around a fibroblast clump is delayed if we increase the excitability inside the clump and around it by increasing the conductance of I_{Na} (G_{Na}) by 1.1 times. The wave distortion occur at 17.7s, which is higher than the averaged value of 15.4 s in the case of normal excitability. For this video, we use 10 frames per second with each frame separated from the succeeding frame by 20ms in real time.

Video S4: **Re-entry via the TP protocol.** Video showing the initiation of reentry, because of our high-frequency pacing (TP protocol), in a medium with a fibroblast clump with R=2.4 cm and p_f=33% (left panel) and 43% (right panel). For this video, we use 10 frames per second with each frame separated from the succeeding frame by 20ms in real time.

Video S5: **TP-pacing induced reentry in our 3D simulations with a cylindrical fibroblast clump.** Video showing the formation of reentry, via our TP stimulation protocol with PCL= 152 ms, in a 3D domain with a cylindrical fibroblast clump, p_f= 65%, and R=2.4 cm. For this video, we use 10 frames per second with each frame separated from the succeeding frame by 20ms in real time.
